# Hypertrophy of the Spinalis Cervicis Muscle in Cervical Dystonia

**DOI:** 10.5334/tohm.811

**Published:** 2023-11-07

**Authors:** Sebastian Loens, Tobias Boppel, Tobias Bäumer

**Affiliations:** 1 Institute of Systems Motor Science, University of Lübeck, Lübeck, Germany; 2 Department of Rare Diseases, University Hospital Schleswig Holstein, Lübeck, Germany; 3 Department of Neuroradiology, University Hospital Schleswig Holstein, Lübeck, Germany

**Keywords:** Cervical dystonia, Botulinum toxin, MRI

## Abstract

**Background:**

Botulinum neurotoxin A (BoNT) is the first line treatment for cervical dystonia (CD) and treatment outcome significantly depends on the correct identification of the muscles involved.

**Phenomenology shown:**

In a case with insufficient response to BoNT treatment further work up with magnetic resonance imaging (MRI) of the neck revealed a hypertrophic spinalis cervicis muscle, that is not commonly involved in CD.

**Educational value:**

This highlights the use of MRI for muscle selection in treatment refractory CD cases.

A 45-year-old cervical dystonia (CD) patient with retrocollis, right sided torticollis and left sided laterocollis responded insufficiently to botulinum toxin treatment. By ultrasound, the right longissimus capitis muscle appeared hypertrophic, otherwise no abnormalities were noted. Repeated ultrasound-guided injections of the right sided splenius capitis, longissimus capitis and obliquus capitis inferior muscles partially improved the torticollis component, while the laterocollis component showed only marginal response to injections into the left trapezius, semispinalis and levator scapulae muscles. No effect on the retrocollis was reported. Magnetic resonance imaging (MRI) of the neck then revealed an untypical prominent variant of the spinalis cervicis muscle on the left side (Figure 1). The typically thin spinalis cervicis muscle extends from the spinous processes of vertebrae Th6 – C6 to the spinous processes of vertebrae C4-C2 and is inconstantly present [1]. Bilateral activation erects the spinal column while unilateral contraction bends the spine sideways. It is usually quite small or even absent and not typically treated in cervical dystonia, in contrast to the semispinalis capitis and semispinalis cervicis muscles, which are regular treatment targets [2]. In our patient dystonic activity of the spinalis cervicis muscle was confirmed by electromyography (EMG). Ultrasound guided injection of 20 units incobotulinumtoxin significantly improved dystonic posturing [3]. This demonstrates the possible involvement of the spinalis cervices muscle in the dystonic pattern of CD, which is not part of standard injection schemes and might hence easily be overlooked. The use of neck imaging to identify hypertrophic muscles and improve treatment outcome has been demonstrated previously [4,5]. Our case illustrates how the use of different diagnostic methods, in this case MRI and EMG, can be used to improve the outcome in difficult cases with insufficient BoNT treatment response.

**Figure 1:**
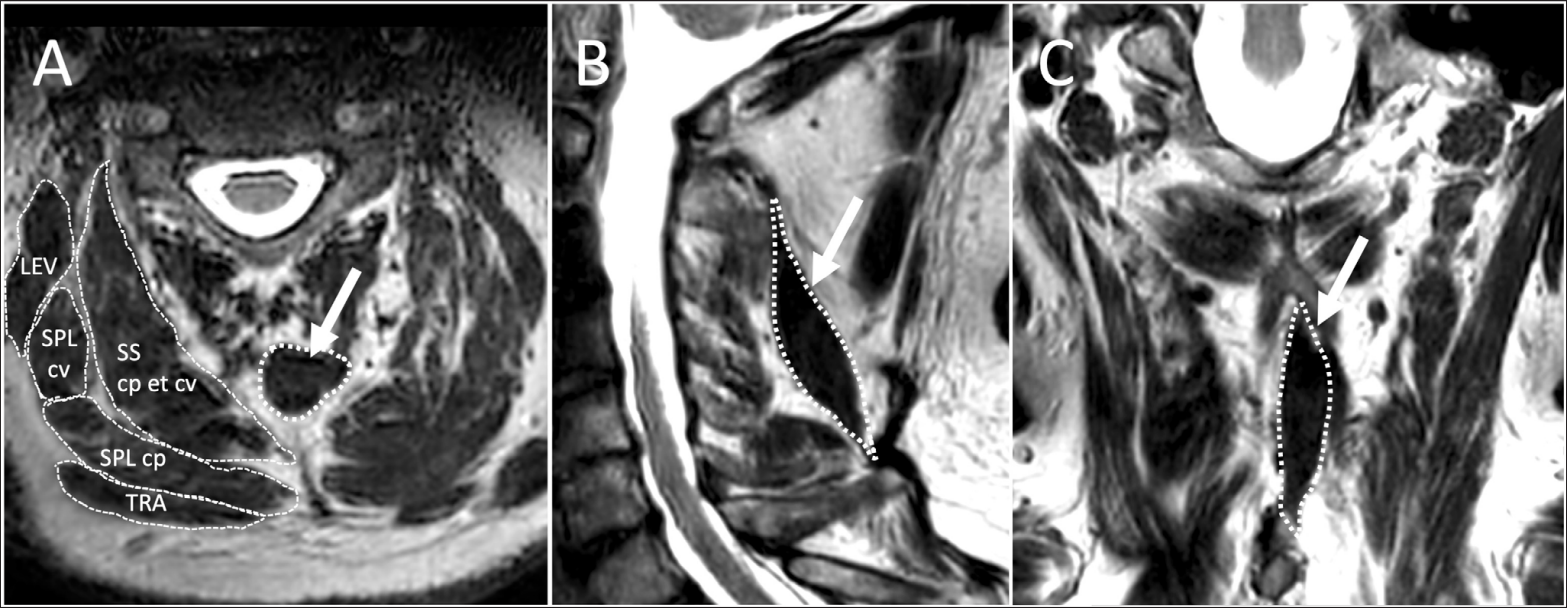
**Neck MRI of a subject with retrocollis.** T2-weighted images depicting the spinalis cervicis muscle (white encircled, arrow); **(A)** Axial and **(C)** coronal images showing the left sided unilateral anlage; **(B)** sagittal image demonstrating its extension from C7 to C2. SScp et cv = semispinalis capitis et cervicis; SPLcp = splenius capitis; SPLcv = splenius cervicis; LEV = levator scapulae; TRA = trapezius.
